# Quality of life among pulmonary hypertension patients in Finland

**DOI:** 10.3402/ecrj.v3.26405

**Published:** 2016-01-18

**Authors:** Merja Kukkonen, Airi Puhakka, Maija Halme

**Affiliations:** 1 Department of Pulmonary Diseases, Helsinki University Central Hospital, Helsinki, Finland; 2 Actelion Pharmaceuticals Sverige AB filial Finland, Espoo, Finland

**Keywords:** PH, PAH, QOL, IPAH, APAH, CTEPH

## Abstract

**Background:**

The purpose of the study was to examine pulmonary hypertension (PH) patients’ quality of life (QOL) for the first time in Finland.

**Methods:**

This was a non-interventional, cross-sectional study. The SF-36v2 questionnaire was sent to the PH patients who had been referred to or followed up on at the Helsinki University Central Hospital's pulmonary clinic for idiopathic pulmonary arterial hypertension, associated pulmonary arterial hypertension (APAH), or chronic thromboembolic PH (CTEPH). The patients were on pulmonary arterial hypertension (PAH) – specific drugs, were at least 18 years old, and had signed an informed consent.

**Results:**

There were 62 patients who fulfilled the inclusion criteria, and 53% of respondents rated their health as moderate. Similarly, 55% of respondents rated their health status approximately the same compared to their situation 1 year ago. QOL was impaired in all other subscales, except for the mental health and mental component score. A majority of patients suffered from PH symptoms, which worsened their QOL. The greatest impact on their QOL was associated with a high World Health Organization (WHO) functional class (FC), poor performance in a 6-min walking test (6MWT), symptoms, oxygen therapy, elevated pro-brain natriuretic peptide, pericardial effusion, APAH etiology, and being retired from work.

**Conclusions:**

The respondents had a reduced QOL, compared to the general population, in all other subscales, except for mental health. APAH patients had the worst QOL. Good results in functional capacity measures (WHO FC, 6MWT) were associated with a better QOL. Patients’ QOL can be improved by reducing the symptoms of PAH.

Pulmonary hypertension (PH) is a condition where the mean pulmonary artery pressure (mPAP) is increased ≥ 25 mmHg, measured by right-heart catheterization (1). Five groups of disorders that cause PH have been identified (2). Pulmonary arterial hypertension (PAH) is further defined by an increase in mPAP≥25 mmHg, a wedge pressure less than 15 mmHg, and pulmonary vascular resistance (PVR)>3 Wood units (WU) (1). PAH is a rare and potentially life-threatening disease. Diagnosis is often only achieved at a late stage of the disease (3).

Treatment and diagnostic practices of PAH and chronic thromboembolic PH (CTEPH) have developed over the last decade in Finland in accordance with international treatment recommendations (1, 4). There are treatment options available from all three pharmacological groups of PAH-specific medications (endothelin receptor antagonists, phosphodiesterase-5 inhibitors, and prostanoids). These drugs can be administered either orally, via inhalation, or by subcutaneous/intravenous infusion, and several medications might be used as combination therapy. The decision on the treatment depends on the disease stage and symptoms (4, 5).

Quality of life (QOL) among PH patients has been examined in numerous studies (6–12), but this is the first study to take place in Finland. As PAH is an orphan disease, the number of patients in QOL studies has been quite limited, reaching approximately 150. In previous PH studies, investigators have used either general measures, such as the Medical Outcomes Study Short Form-36 (SF-36) in both versions 1 and 2, the Nottingham Health Profile, European Quality of Life 5 Dimensions (6–13), or PH-specific measures such as the Cambridge Pulmonary Hypertension Outcome Review (CAMPHOR) (10, 11), Minnesota Living with Heart Failure – PH-specific version (8–10), and Living with Pulmonary Hypertension questionnaire (13). Furthermore, disease-specific questionnaires for diseases other than PH have also been used, such as the Chronic Heart Failure Questionnaire (14) and the obstructive pulmonary disease – specific St. George's Respiratory Questionnaire (SGRQ) (7).

There are also new PH-specific QOL tools available or underway, such as the emPHasis-10 tool for easy and rapid use in clinical settings and the PAH-SYMPACT tool for clinical trials (15–18).

SF-36 is a general, non-disease-specific QOL instrument (19). It is considered to be a useful tool in PH patients and treatment evaluation. It correlates well with the 6-min walking test (6MWT) and World Health Organization (WHO) class, but not with hemodynamic measurements (8). It has also been suggested that health-related QOL (HRQOL) is associated with prognosis in PAH (12).

The outcomes from previous studies show that PAH patients’ QOL is deteriorated on the subscales of general health, physical functioning, physical role functioning, and vitality when measured with the SF-36v2 (11, 20). Several background factors that have an impact on this were identified; worse QOL was associated with fatigue, weakness, and abdominal discomfort (9), as well as functional ability, level of education, the use of oxygen, the time of diagnosis, and the use of calcium channel blockers (11). The frequency of symptoms can have an impact on the QOL in PAH patients. Severe cardiopulmonary symptoms were associated with a worse QOL (20).

Because no previous local data were available, we decided to explore the QOL among PH patients in Finland in an observational, cross-sectional study.

## Methods

During the spring of 2012 the QOL questionnaire was sent to 93 patients who were at least 18 years old and who had been examined or followed up on for idiopathic pulmonary arterial hypertension (IPAH), associated pulmonary arterial hypertension (APAH), or CTEPH at the Helsinki University Central Hospital, Department of Pulmonary Diseases, since the year 2000. The patients were sent a cover letter, patient information leaflet, informed consent form, background information sheet, the SF-36v2 QOL form, and a return envelope. Partially completed forms were also accepted in the analysis.

The background information that was collected included the age, gender, mode of living, employment status, medication, symptoms, 6MWT, and surgical procedures for treatment of PH. The medical records were reviewed for the latest available background data (6MWT, N-terminal pro-brain natriuretic peptide (pro-BNP), echocardiographic parameters such as the right atrial and ventricular total surface area, tricuspid annular plane systolic excursion, pericardial effusion count, right-heart catheterization parameters, such as mPAP PVR, disease etiology classification and medications). The WHO functional class (FC) was estimated based on a question on the SF-36 form (how much a moderately strenuous activity e.g. brisk walking on level ground is restricted by health issues).

The SF-36v2 method was chosen, as it was commercially available in both of the two official languages in Finland – Finnish and Swedish. With 36 questions, it measures eight physical and mental health areas, as well as physical and mental component summary scores. For norm-based scoring, every area has the same mean (50) and standard deviation (10). It is based on tests of the normal US population in 1998. The subscale and component summary scores usually range between 20 and 70. If an individual respondent's scores are less than 45 or if the group's mean is less than 47, it is considered to be lower, compared to the healthy population (21).

The scores were calculated electronically with SF-36 software and analyzed with SPSS (SPSS Inc., Chicago, IL, USA) statistical software. Results are expressed as the mean and the standard deviation. Continuous variables were analyzed with one-way analysis of variance (ANOVA) and with Bonferroni-corrected post-hoc tests. The Brown-Forsythe and Welch tests were examined when the equal variance assumption was not valid.

The study protocol was approved by the Ethics Committee of Helsinki University Central Hospital.

## Results

The response rate was 84% (78/93). The final analysis consisted of 62 patients who fulfilled the inclusion criteria. Ten patients were excluded from the study as they did not have PAH-specific medication (one IPAH, one APAH, seven CTEPH, and one unspecific heart disease). In addition, six patients were excluded because their diagnosis did not meet the inclusion criteria (two pulmonary veno-occlusive diseases, three sleep apneas, and one recurrent acute pulmonary embolism), although three of them used PAH-specific medication.

Background information is presented in Table 1.

**Table 1:** Patient characteristics

Patient characteristics	*n* (%)	Mean±SD
Age		53±16.2
Gender, female	46 (74%)	
Living arrangements		
• Single	28 (45%)	
• With a family member	34 (55%)	
Employment status		
• Unemployed	3 (5%)	
• Employed	17 (27%)	
• On sick leave	3 (5%)	
• Retired	39 (63%)	
Etiology		
• IPAH	25 (40%)	
• APAH	16 (26%)	
• CTEPH	21 (34%)	
WHO FC		
• I	8 (13%)	
• II	28 (45%)	
• III	26 (42%)	
Medications		
• PDE-5i	55 (89%)	
• ERA	19 (31%)	
• Prostanoids	12 (19%)	
• CCBs	15 (24%)	
• Warfarin/LMWH	46/11 (74/18%)	
• Digoxin	14 (23%)	
• Diuretics	31 (50%)	
• Oxygen	8 (13%)	
6MWT (*n*=59)		443 m±160
Pro-BNP (*n*=59)		1,105 ng/l±1,739
Right heart surface (*n*=59)		51 cm^2^±21
TAPSE (*n*=59)		18 mm±6
mPAP (*n*=60)		49 mmHg±9
PVR (*n*=35)		11 WU±4
Pericardial effusion, yes	7 (11.5%)	

IPAH, idiopathic pulmonary arterial hypertension; APAH, associated pulmonary arterial hypertension; CTEPH, chronic thromboembolic pulmonary hypertension; WHO FC, World Health Organization functional class; PDE5i, phosphodiesterase type 5 inhibitors; ERA, endothelin receptor antagonists; CCBs, calcium channel blockers; LMWH, low-molecular-weight heparins; 6MWT, 6-min walking test; pro-BNP, pro-brain natriuretic peptide; TAPSE, tricuspid annular plane systolic excursion; mPAP, mean pulmonary artery pressure; PVR, pulmonary vascular resistance.

**Fig. 1:**
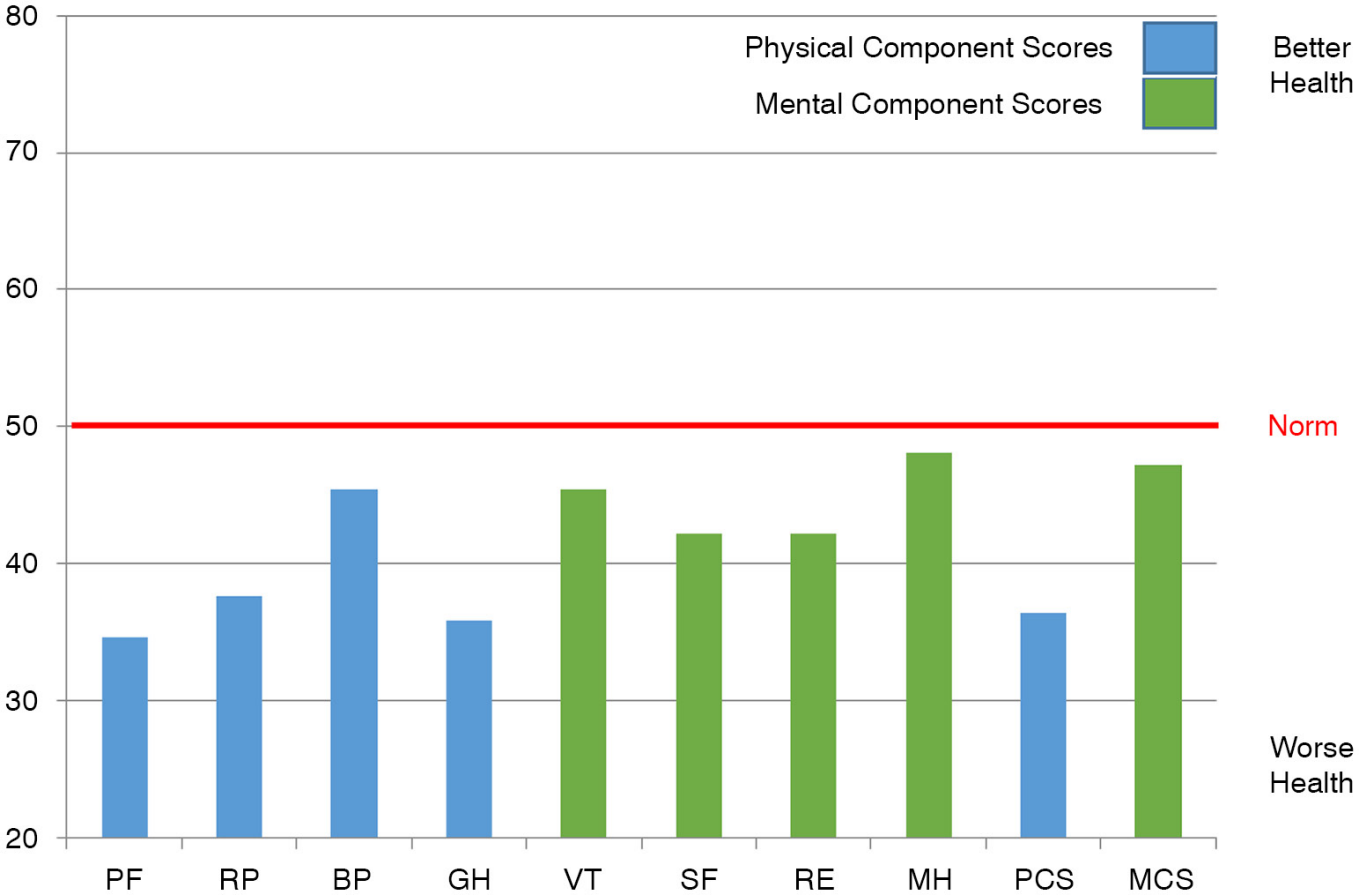
Scores for total sample. PF, physical functioning; RP, physical role functioning; BP, bodily pain; GH, general health; VT, vitality; SF, social functioning; RE, emotional role functioning; MH, mental health; PCS, physical component score; MCS, mental component score.

Of the respondents, 53% assessed their health as moderate, 39% as good or very good, and 8% as poor. Compared to the situation 1 year ago, 55% of the respondents reported their state of health as roughly the same, 21% as somewhat or much better, and 24% as somewhat or a lot worse. All of the respondents had a reduced QOL, compared to the general population, in all other subscales, except for mental health and mental component summary (Fig. 1). There were no statistically significant differences between genders.

In WHO FC III patients, QOL was significantly impaired on the subscales of physical functioning, physical role functioning, general health, social functioning, and physical component summary score, compared with WHO FC II (all *p*<0.006). However, the only significant difference between the WHO FC I and II was found on the physical function scale (*p*=0.002). The QOL was significantly reduced with patients whose 6MWT result was under 330 meters, compared to the group with a result over 330 meters. There was a significant difference between the 6MWT groups in physical functioning, physical role functioning, and the physical component summary score (*p*<0.001).

The etiology of PAH was associated with QOL. The APAH patients had the worst QOL. Their physical functioning was significantly impaired, compared with IPAH patients (*p*=0.008). Compared with both IPAH and CTEPH patients, the other significant differences were found in physical role functioning (*p*=0.008, *p*=0.022, respectively), general health (*p*=0.011, *p*=0.013), as well as the physical component summary score (*p*=0.009, *p*=0.023).

A significantly better QOL was observed in 35–54-year-old patients, compared to patients over 55 years, in physical role functioning (*p*=0.028). In contrast, for mental health their QOL was significantly worse (*p*=0.003), compared to the older group. The QOL of retired patients was significantly reduced, compared to employed patients, in physical functioning, physical role functioning, and in the physical component summary score (*p*=0.022, *p*=0.009, *p*=0.010, respectively).

The symptoms worsened the QOL. Respiratory symptoms were associated with significantly lower scores on vitality and mental component summary scores (*p*=0.014, *p*=0.040, respectively). Patients with edema had lower scores on the subscales of physical functioning, physical role functioning, general health, and physical component summary scores (*p*=0.019, *p*=0.030, *p* < 0.001, *p*=0.001, respectively). Fatigue symptoms were associated with a lower physical component summary score (*p*=0.048). The heart-related symptoms were associated with bodily pain, general health, vitality, and social functioning (*p* < 0.001, *p*=0.043, *p*=0.012, *p*=0.045, respectively). Vertigo was associated with lower scores on mental health and the mental component summary (*p*=0.025, *p*=0.041, respectively). Other symptoms worsened the QOL on physical role functioning, general health, social functioning, and physical component summary scores (*p*=0.036, *p*=0.020, *p*=0.019, *p*=0.030, respectively). Oxygen therapy was associated with worse QOL, physical functioning, and physical role functioning, as well as physical component summary score (*p*=0.010, *p*=0.012, *p*=0.013, respectively).

Patients with pro-BNP values over 1,800 had significantly worse physical functioning (*p*=0.010) and physical role functioning (*p*=0.040) scores, compared with the patients with pro-BNP values less than 221. Patients with pericardial fluid had impaired physical role functioning, social functioning, mental health, and mental component summary scores (*p*=0.029, *p*=0.018, *p*=0.024, *p*=0.03, respectively).

Gender, mode of living, and time since diagnosis did not have an effect on the QOL. There was no statistical difference between specific drug treatment groups. The patients were divided into three mPAP groups as follows: mild (mPAP) 25–35 mmHg (*n*=5), moderate 35.1–45 mmHg (*n*=20), or severe >45 mmHg (*n*=35). The mean physical component score (PCS) was 38, 35, and 38; and the mean mental component score (MCS) was 50, 48, and 46 in the mild, moderate, and severe mPAP groups, respectively. The PVR groups were ≤8.76 WU (*n*=10), 8.77–12 WU (*n* = 11), and ≥ 12.1 WU (*n*=14). The mean PCS was 34, 35, and 37 and the mean MCS was 48, 50, and 48 in the mild, moderate, and severe PVR groups, respectively. Pulmonary resistance and mean pulmonary arterial pressure were not statistically significantly associated with QOL.

## Discussion

Our study is the first description of PH patients’ QOL in Finland. As background, the prevalence of IPAH in Finland was 5.8 cases/million in 2005, and in the more recent Finnish study the PAH prevalence was 21.5/million (22, 23). It was estimated that there were 165 patients with PAH and CTEPH in Finland (23). Reflecting on these prevalence figures and comparing them to international PH HRQOL studies, where the patient number has been about 155, our patient number (62) was satisfactory and the response rate (84%) was good. A significant proportion of Finnish patients with PAH have been referred to or followed up on at Helsinki University Central Hospital. Considering this, the current study gave quite a comprehensive view on the QOL in the Finnish PH patient population.

There were some limitations to our study. Some patients were excluded from the study because they were not treated with PAH-specific drugs. This was the case, for example, for CTEPH patients, whose first-line treatment option is potentially curative surgery, specifically pulmonary endarterectomy. A majority of these excluded patients without medication were in the process of evaluation for operability or on the waiting list for the surgery. The inclusion criteria of PAH-specific medication, combined with a diagnosis of IPAH, APAH, or CTEPH, was set in order to ensure selection of PAH – and inoperable CTEPH – patients from the wide PH patient population. These inclusion criteria might of course have led to the exclusion of patients with very mild disease. However, there was only one mild IPAH patient excluded from the study based on these specific medication criteria; thus it was not a major issue.

Statistical analysis was also limited by the fact that the test set was small and the subgroups even smaller. However, it is important to gain information, in order to identify any developmental needs in treatment practices. This study provided new descriptive information about PH patients’ QOL in Finland.

There are two HRQOL tools available in Finnish and Swedish, SF-36v2 and SGRQ. SGRQ was originally developed for the assessment of chronic obstructive pulmonary disease and is not a PH-specific tool. The PAH-specific tool CAMPHOR is available in Swedish, but it is unfortunately not available in Finnish. We chose the SF-36v2, since it was frequently used in PAH studies and was commercially available in both Finnish and Swedish.

In our study, QOL was impaired in all subscales, except for mental health. The mental health and mental component summary scores in the total material were surprisingly good. However, among those of working age, mental health QOL was worse than in the group 55 years of age and older. The reason for this is unknown, but it can be speculated that chronic disease causes more limitations in life for patients of working age, compared to their healthy peers, than in older groups, and thus patients of working age see their QOL as being worse. In previous studies, neither age (7, 9) nor work status was associated with HRQOL in PH (11). Being retired mainly deteriorated QOL in the physical dimensions in our study.

QOL was significantly influenced by the WHO FC, 6MWT, the symptoms of the disease, oxygen therapy, pro-BNP, the presence of pericardial effusion, being retired, and disease etiology. Specific drug therapy, mode of living, gender, diagnosis year, and PVR and mPAP had no effect on QOL. Not surprisingly, WHO FC III, and shorter 6MWT result were associated with worse QOL. The patients’ WHO FC was not always found in the medical records, so it was based on their answers about physical activity in the questionnaire. Since nobody reported dyspnea at rest, it was possible that the WHO FC IV patients were not identified properly. It is surprising that poor physical performance did not appear to be related to worse QOL on the mental subscales (with the exception of social functioning between WHO FC II versus WHO FC III). This might be a reflection of a successful overall treatment organization in Finland including patient follow-up in dedicated PAH centers and access to PAH-specific medicines supported by the government.

APAH patients had a significantly reduced QOL, compared with both IPAH and CTEPH patients. A majority (*n*=10) of the APAH patients had a connective tissue disease. The rest of the patients had either congenital heart disease or their PAH was associated with drug or toxin use. The background disease might be especially complicated and disabling in PAH associated with systemic sclerosis. Similar results have also been obtained in other studies (7, 9).

Elevated pro-BNP, as well as the presence of pericardial effusion, was associated with worse QOL. Both findings suggest a progression of the disease and right heart failure, so the result was expected. Disease-related symptoms worsened QOL. On this basis it might be a reasonable treatment goal to reduce symptoms, as this could also improve QOL.

Because the data were collected retrospectively, there were no recent hemodynamic measurements available. Thus, a limitation of the study is that mPAP and PVR results did not properly reflect the situation at the time of the QOL data collection, and some of the results were missing from the medical records of the study center, as the diagnostic right-heart catheterization had been done earlier by the referring hospital. However, keeping these facts in mind, in our study, mPAP and PVR were not associated with the QOL subscales. This is in line with earlier reports (7).

It would be interesting to see QOL before starting the medication and after regular intervals on treatment (e.g. 3 months, 1 year) in future longitudinal studies.

## Conclusions

This is the first time the QOL of PH patients has been evaluated in Finland. QOL was reduced, compared with the normal population, in all areas, except for mental health. Patient QOL can be improved by reducing the symptoms of PAH. The QOL questionnaire provided significant information about patients’ treatment needs.
